# Genomic copy number gains of ErbB family members predict poor clinical outcomes in glioma patients

**DOI:** 10.18632/oncotarget.21228

**Published:** 2017-09-23

**Authors:** Rui Liu, Yiping Qu, Lihong Chen, Jun Pu, Sharui Ma, Xiaozhi Zhang, Qi Yang, Bingyin Shi, Peng Hou, Meiju Ji

**Affiliations:** ^1^ Department of Endocrinology, The First Affiliated Hospital of Xi’an Jiaotong University School of Medicine, Xi’an 710061, P. R. China; ^2^ Department of Radio-Oncology, The First Affiliated Hospital of Xi’an Jiaotong University School of Medicine, Xi’an 710061, P. R. China; ^3^ Department of Critical Care Medicine, The First Affiliated Hospital of Xi’an Jiaotong University, Xi’an 710061, P. R. China; ^4^ Key Laboratory for Tumor Precision Medicine of Shaanxi Province, The First Affiliated Hospital of Xi’an Jiaotong University, Xi’an 710061, P. R. China; ^5^ Center for Translational Medicine, The First Affiliated Hospital of Xi’an Jiaotong University, Xi’an 710061, P.R. China

**Keywords:** glioma, ErbB family, copy number gain, clinical outcomes

## Abstract

The aim of this study was to investigate copy number of ErbB family members (including *EGFR*, *HER2*, *HER3* and *HER4*) in a cohort of gliomas and benign meningiomas (control subjects), and explore the associations of their copy number with clinicopathological characteristics and clinical outcomes of glioma patients. Using real-time quantitative PCR assay, we demonstrated that copy number of *EGFR*, *HER2*, *HER3* and *HER4* in glioma patients was significantly increased compared to control subjects. Moreover, our data also showed that the risk of cancer-related death was positively associated with copy number gain (CNG) of *EGFR, HER3* and *HER4*, but not *HER2*. CNG of *EGFR* and *HER2* was positively related to radiotherapy, while CNG of *HER3* and *HER4* was negatively related to chemotherapy. Importantly, *EGFR* CNG significantly shortened median survival times of glioma patients regardless of gender, tumor grade and therapeutic regimens. Stratified analysis showed that CNG of *HER2*-*4* almost did not influence the survival of male patients, patients with high-grade tumors and patients receiving chemotherapy, but dramatically shortened median survival times of female patients, those with low-grade tumors and those receiving radiotherapy. Collectively, our data not only demonstrate that the members of ErbB family are frequently amplified in gliomas, but also suggest that these common genetic events may be prognostic factors for poor clinical outcomes in glioma patients.

## INTRODUCTION

Gliomas, also named as gliocytoma, are the most common primary brain tumors in the central nervous system (CNS). It accounts for about 80% of all brain tumors, with an incidence rate of approximately 7 per 100,000 worldwide [1]. The patients with gliomas may have several neurological symptoms such as headaches, seizures, memory loss, vomiting and visual changes [2]. Despite the existence of various treatments, including surgery, chemotherapy or radiotherapy, its prognosis is still poor [3, 4]. Therefore, determining the genetic alterations of classic oncogenes will improve the accuracy of clinical interpretations and the effectiveness of therapeutics for this cancer.

Tumors are thought to originate from a single clonal state that then expands clonally, accompanied by genetic events that give rise to functional differences, resulting in different stages and characteristics of neoplastic development [5]. Copy number variation (CNV) is increasingly linked to the genetic and phenotypic diversity among cancers, and is frequently associated with the activation of oncogenic drivers or the deletion of tumor suppressors [6–8]. Like other malignancies, the current studies have shed light on molecular events of oncogenes and tumor suppressor genes in gliomas due to numerical chromosomal abnormalities such as genomic gains and losses [9, 10].

Growth factors mediate diverse biologic responses including cell proliferation, migration, differentiation and survival by binding to and activating cell-surface receptors with receptor tyrosine kinases (RTKs) activity [11, 12]. Epidermal growth factor (EGF), one of the first growth factors, was discovered in the early 1960s. EGFs were shown high affinity to bind with its specific receptor EGFR [13]. The EGFR receptor tyrosine kinases family consists of ErbB1 (also known as EGFR or HER1), ErbB2/HER2, ErbB3/HER3 and ErbB4/HER4 [14]. Genomic alterations such as copy number gain (CNG) in members of ErbB family generally lead to their activation, thereby stimulating downstream intracellular signaling pathways, including the RAS/RAF/MEK/ERK (MAPK/ERK), PI3K/AKT, and JAK/STAT pathways, which promote cell proliferation, survival and migration [15]. Given that aberrant activation of ErbB receptors is frequently observed in multiple types of cancer, clinical applications have rapidly emerged around attempts to therapeutically inhibit the function of ErbB receptors in different cancers including monoclonal antibodies (mAbs), small-molecule TKIs and other agents like peptides, affibodies, nanobodies, etc [16, 17]. However, the prognostic significance of CNG of members of ErbB family in gliomas remains largely unclear.

In this study, we investigated copy number of members of ErbB family by using quantitative PCR (qPCR) approach in a cohort of gliomas and control subjects, and explored the association of CNG of members of ErbB family with clinical outcomes of glioma patients.

## RESULTS

### Frequent CNG of members of ErbB family in glioma patients

**Figure 1:**
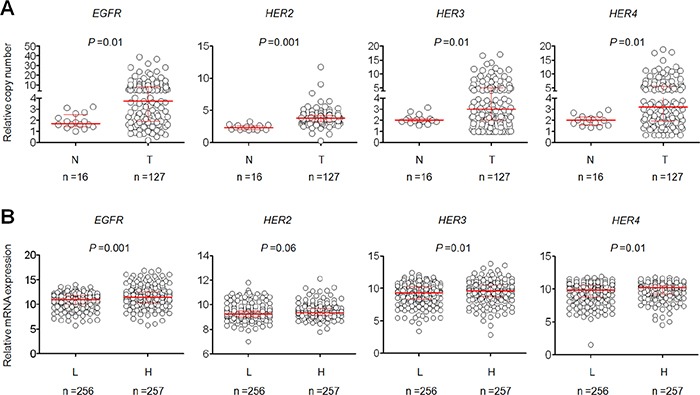
Copy number of members of ErbB family in a cohort of gliomas and control subjects **(A)** Copy number of *EGFR*, *HER2*, *HER3* and *HER4* of each case was determined by a qPCR assay. Each circle represents copy number of the indicated gene of an individual case. Horizontal lines indicate median and interquartile range. T: tumor tissues; N: control subjects. **(B)** The relationship between copy number of the indicated gene and its mRNA expression in low-grade gliomas from The Cancer Genome Atlas (TCGA) dataset. Horizontal lines indicate median and interquartile range. “L” and “H” represent low- and high-copy number of the indicated gene, respectively. A median copy number of each indicated gene is used as cut-off value.

Copy number of ErbB family members was examined in a cohort of 127 gliomas and 16 benign meningiomas as control subjects using qPCR approach. As shown in Figure 1A and Supplementary Table 1, glioma patients exhibited a significantly higher copy number of *EGFR* (6.53 *vs*. 1.91; *P* =0.01), *HER2* (3.87 *vs*. 2.36; *P* =0.001), *HER3* (4.21 *vs*. 2.09; *P* =0.01), and *HER4* (4.43 *vs*. 1.98; *P* =0.01) than control subjects. When a copy number of ≥4 of the indicated genes was considered as CNG (or gene amplification), we found that *EGFR* CNG in 62/127 (48.8%) gliomas, *HER2* in 44/127 (34.6%) gliomas, *HER3* in 47/127 (37.0%) gliomas, and *HER4* in 50/127 (39.4%) gliomas, whereas none was found in control subjects (Table 1). Also shown in Table 1, *IDH1* mutations were found in 45 of 127 (35.4%) patients. Given that genomic alterations are different among the subgroups of glioma, we further compared copy number of ErbB family members according to tumor grade. As shown in Supplementary Figure 1, copy number of *EGFR* and *HER2* was not different between low-grade gliomas (LGGs) and high-grade gliomas (GBMs); however, copy number of *HER3* and *HER4* was significantly increased in LGGs as compared to GBMs. In addition, two or more amplification events were found in 57 of 127 (44%) patients (Supplementary Figure 2). To test the association of copy number of the above genes and their mRNA levels, we analyzed the corresponding data in a total of 513 low-grade gliomas using The Cancer Genome Atlas (TCGA) dataset from the Cancer Browser database (https://genome-cancer.soe.ucsc.edu). We divided these cases into low- and high-copy groups by using median copy number of each gene. As shown in Figure 1B, the mRNA levels of these four genes in high-copy group were higher than low-copy group, especially in *EGFR* (*P* <0.001).

**Table 1:** Clinicopathological characteristics of glioma patients

Characteristics	No. of patients (%)	No. of *EGFR* CNG (%)	No. of *HER2* CNG (%)	No. of *HER3* CNG (%)	No. of *HER4* CNG (%)	No. of *IDH1* mutations (%)
Gender						
Male	69 (54.3)	32 (46.4)	22 (31.9)	28 (40.6)	28 (40.6)	24 (34.8)
Female	58 (45.7)	30 (51.7)	22 (37.9)	19 (32.8)	22 (37.9)	21 (36.2)
Age, years						
Mean	45.1	n/a	n/a	n/a	n/a	n/a
SD	16.7	n/a	n/a	n/a	n/a	n/a
WHO grade						
I	17 (13.4)	11 (64.7)	5 (29.4)	10 (58.8)	10 (58.8)	4 (23.5)
II	61 (48.0)	28 (45.9)	24 (39.3)	23 (37.7)	24 (39.3)	29 (47.5)
III	34 (26.8)	16 (47.1)	7 (20.6)	11 (32.4)	14 (41.2)	11 (32.4)
IV	15 (11.8)	7 (46.7)	8 (53.3)	3 (20.0)	2 (13.3)	1 (6.7)
KPS score						
≤80	51 (40.2)	25 (49.0)	22 (43.1)	18 (35.3)	18 (35.3)	17 (33.3)
>80	76 (59.8)	37 (48.7)	22 (28.9)	29 (38.2)	32 (42.1)	28 (36.8)
Recurrence						
No	31 (24.4)	9 (29.0)	9 (29.0)	7 (22.6)	9 (29.0)	14 (45.2)
Yes	96 (75.6)	53 (55.2)	35 (36.5)	40 (41.7)	41 (42.7)	31 (32.3)
Radiotherapy						
No	48 (37.8)	20 (41.7)	12 (25.0)	15 (31.2)	16 (33.3)	14 (29.2)
Yes	79 (62.2)	42 (53.2)	32 (40.5)	32 (40.5)	34 (43.0)	31 (39.2)
Chemotherapy						
No	76 (59.8)	39 (51.3)	26 (34.2)	31 (40.8)	35 (46.1)	26 (34.2)
Yes	51 (40.2)	23 (45.1)	18 (35.3)	16 (31.4)	15 (29.4)	19 (37.3)
Survival status						
Dead	75 (59.1)	47 (62.7)	28 (37.3)	34 (45.3)	35 (46.7)	16 (21.3)
Alive	52 (40.9)	15 (28.8)	16 (30.8)	13 (25.0)	15 (28.8)	29 (55.8)
Smoking						
No	92 (72.4)	44 (47.8)	36 (39.1)	30 (32.6)	34 (37.0)	33 (35.9)
Yes	35 (27.6)	18 (51.4)	8 (22.9)	17 (48.6)	16 (45.7)	12 (34.3)
Drinking						
No	121 (95.3)	61 (50.4)	43 (35.5)	45 (37.2)	49 (40.5)	43 (35.5)
Yes	6 (4.7)	1 (16.7)	1 (16.7)	2 (33.3)	1 (16.7)	2 (33.3)
Epilepsy						
No	70 (55.1)	38 (54.3)	26 (37.1)	28 (40.0)	28 (40.0)	13.8 (18.6)
Yes	57 (44.9)	24 (42.1)	18 (31.6)	19 (33.3)	22 (38.6)	32 (56.1)

### Association of CNG of members of ErbB family with clinicopathological features in glioma patients

Given frequent CNG of ErbB family members in gliomas, the associations of their CNG with clinicopathological features of glioma patients were then investigated in a cohort of glioma patients. When copy number of ≥4 was defined as CNG, the glioma patients were subsequently categorized into CNG- and non-CNG-groups of the indicate genes. As shown in Table 2, by using univariate regression analysis, we found that the risk of cancer-related death was significantly increased by the presence of *EGFR* CNG (OR =4.14, 95% CI =1.94-8.86, *P* <0.001), *HER3* CNG (OR =2.48, 95% CI =1.15-5.40, *P* =0.02) and *HER4* CNG (OR =2.16, 95% CI =1.02-4.58, *P* =0.04). The patients with *EGFR* CNG had a much higher risk of tumor recurrence (OR =3.01, 95% CI =1.26-7.21, *P* =0.01). *HER2* was more likely to be amplified in young patients (OR =0.61, 95% CI =0.40-0.93, *P* =0.02). Notably, *HER3* and *HER4* CNG were significantly negatively associated with tumor grade (the former: OR =0.61, 95% CI=0.39-0.95, *P* =0.03; the latter: OR =0.66, 95% CI =0.40-0.95, *P* =0.03). Increasing evidences have demonstrated that *IDH1* mutations are important genetic events, and closely associated with survival benefit in gliomas [18, 19]. Our data showed that the association of low copy number of *EGFR* with *IDH1* mutations (Table 2). In addition, we did not find significant correlations between CNG of members of ErbB family and other clinicopathological characteristics such as gender, KPS score, radiotherapy, chemotherapy, smoking, drinking and epilepsy (Table 2).

**Table 2:** CNG of members of ErbB family in gliomas: univariate associations with clinicopathological characteristics

Characteristics	*EGFR* CNG		*HER2* CNG		*HER3* CNG		*HER4* CNG	
	OR^*^ (95% CI)	*P*	OR^*^ (95% CI)	*P*	OR^*^ (95% CI)	*P*	OR^*^ (95% CI)	*P*
Gender	1.24 (0.62-2.49)	0.59	1.31 (0.63-2.72)	0.48	0.71 (0.34-1.48)	0.36	0.90 (0.44-1.83)	0.76
Age^1^	1.27 (0.86-1.88)	0.23	0.61 (0.40-0.93)	0.02	0.95 (0.63-1.41)	0.79	0.98 (0.66-1.46)	0.93
WHO grade^2^	0.84 (0.56-1.27)	0.42	1.08 (0.71-1.66)	0.71	0.61 (0.39-0.95)	0.03	0.66 (0.40-0.95)	0.03
KPS score^3^	0.98 (0.49-2.00)	0.97	0.54 (0.26-1.13)	0.10	1.13 (0.54-2.37)	0.74	1.33 (0.64-2.78)	0.44
Recurrence	3.01 (1.26-7.21)	0.01	1.40 (0.58-3.38)	0.45	2.45 (0.96-6.24)	0.06	1.82 (0.76-4.37)	0.16
Radiotherapy	1.59 (0.77-3.28)	0.21	2.04 (0.92-4.51)	0.08	1.50 (0.70-3.20)	0.30	1.51 (0.72-3.19)	0.28
Chemotherapy	0.78 (0.38-1.59)	0.49	1.05 (0.49-2.20)	0.90	0.66 (0.31-1.40)	0.28	0.48 (0.23-1.03)	0.06
Survival status^4^	4.14 (1.94-8.86)	<0.001	1.34 (0.63-2.84)	0.45	2.48 (1.15-5.40)	0.02	2.16 (1.02-4.58)	0.04
Smoking	1.16 (0.53-2.52)	0.72	0.46 (0.19-1.12)	0.08	1.95 (0.88-4.32)	0.10	1.44 (0.65-3.16)	0.37
Drinking	0.20 (0.02-1.71)	0.14	0.36 (0.04-3.20)	0.36	0.84 (0.15-4.80)	0.85	0.29 (0.03-2.59)	0.27
Epilepsy	0.61 (0.30-1.24)	0.17	0.78 (0.37-1.64)	0.51	0.75 (0.36-1.56)	0.44	0.94 (0.46-1.93)	0.87
*IDH1* mutations	0.32 (0.15-0.69)	0.004	1.06 (0.49-2.28)	0.87	0.78 (0.37-1.67)	0.53	0.90 (0.43-1.90)	0.79

^*^ OR: odds ratio with 95% confidence interval; ^1^Age (per 20 years); ^2^WHO grade (I, II, III and IV); ^3^KPS (>80; ≤80); ^4^Survival status (alive *vs*. dead). The cases < 4 copies were used as reference.

Next, multivariable regression analysis was conducted to assess the independent associations of CNG of the above genes with tumor grade, recurrence, radiotherapy, chemotherapy and survival status in glioma patients. Similar to the findings from univariate analysis, CNG of *EGFR* (OR =9.61, 95% CI =2.83-32.7, *P* <0.001), *HER3* (OR =4.47, 95% CI =1.35-14.75, *P* =0.01) and *HER4* (OR =5.07, 95% CI =1.50-17.06, *P* =0.009) was still significantly associated with cancer-related death in glioma patients (Table 3). Also shown in Table 3, *EGFR* (OR =0.56, 95% CI =0.33-0.94, *P* =0.03), *HER3* (OR =0.42, 95% CI =0.24-0.72, *P* =0.002) and *HER4* (OR =0.42, 95% CI =0.24-0.73, *P* =0.002) genes were more likely to be amplified in the patients with low-grade tumors as compared to those with high-grade tumors, suggesting that CNG of *EGFR*, *HER3* and *HER4* may be early-stage genetic events in glioma tumorigenesis. Notably, we found that CNG of *EGFR* (OR =2.72, 95% CI =1.12-6.64, *P* =0.03) and *HER2* (OR =2.42, 95% CI =1.03-5.73, *P* =0.04) was more likely to be found in the patients receiving postoperative radiotherapy, whereas CNG of *HER3* (OR =0.39, 95% CI =0.16-0.95, *P* =0.04) and *HER4* (OR =0.28, 95% CI =0.11-0.68, *P* =0.005) was more likely to be found in the patients who did not receive postoperative chemotherapy (Table 3).

**Table 3:** CNG of members of ErbB family in gliomas: multivariate models assessing WHO grade, recurrence, radiotherapy, chemotherapy and survival status

Characteristics	*EGFR* CNG		*HER2* CNG		*HER3* CNG		*HER4* CNG	
	OR^*^ (95% CI)	*P*	OR^*^ (95% CI)	*P*	OR^*^ (95% CI)	*P*	OR^*^ (95% CI)	*P*
WHO grade^1^	0.56 (0.33-0.94)	0.03	1.11 (0.69-4.64)	0.42	0.42 (0.24-0.72)	0.002	0.42 (0.24-0.73)	0.002
Recurrence	0.99 (0.28-3.58)	0.99	1.01 (0.30-3.46)	0.98	1.14 (0.52-2.52)	0.74	1.19 (0.32-4.38)	0.80
Radiotherapy	2.72 (1.12-6.64)	0.03	2.42 (1.03-5.73)	0.04	1.89 (0.77-4.64)	0.16	2.10 (0.86-5.13)	0.10
Chemotherapy	0.44 (0.19-1.05)	0.07	0.87 (0.40-1.93)	0.74	0.39 (0.16-0.95)	0.04	0.28 (0.11-0.68)	0.005
Survival status^2^	9.61 (2.83-32.7)	<0.001	1.56 (0.53-4.64)	0.42	4.47 (1.35-14.75)	0.01	5.07 (1.50-17.06)	0.009

^*^ OR: odds ratio with 95% confidence interval (CI); ^1^WHO grade (I, II, III and IV); ^2^Survival status (alive *vs*. dead). The cases < 4 copies were used as reference.

We further divided the patients into two groups according to gender. As shown in Supplementary Table 2, CNG of *EGFR*, *HER2* and *HER3* was positively associated with tumor recurrence, especially *EGFR* in female patients. Moreover, we also found that CNG of *EGFR*, *HER2*, *HER3* and *HER4* was correlated with the risk of cancer-related death in female patients (Supplementary Table 2). In male patients, our data showed that *HER4* was more likely to be amplified in early-stage tumors. CNG of *HER3* and *HER4* was more likely to be found in the patients who did not receive postoperative chemotherapy, while *EGFR* CNG was more likely to be amplified in the patients receiving radiotherapy (Supplementary Table 3). As expected, we also found that *EGFR* CNG was significantly associated with increased risk of male patients (Supplementary Table 3). Next, multivariable regression analysis demonstrated that *EGFR* CNG in female patients and CNG of *HER3* and *HER4* in male patients were still negatively associated with tumor grade (Supplementary Tables 4 and 5). In addition, we also found that *EGFR* CNG in both female and male patients and CNG of *HER3* and *HER4* in male patients were closely associated with increased risk of cancer-related death (Supplementary Tables 4 and 5). Also shown in Supplementary Table 5, our data indicated that CNG of *HER2*-*4* was more frequently found in male patients receiving postoperative radiotherapy. In contrast, CNG of *HER3* and *HER4* was more frequently observed in male patients who did not receive postoperative chemotherapy. However, clinical significance and exact mechanism responsible for these associations remain be further elucidated.

**Table 4:** Prognostic value of clinicopathological factors and CNG of members of ErbB family using univariate Cox regression analysis

Characteristics	HR^*^ (95% CI)	*P*
*EGFR CNG*	2.31 (1.44-3.69)	0.001
*HER2 CNG*	1.21 (0.75-1.92)	0.44
*HER3 CNG*	1.36 (0.85-2.15)	0.19
*HER4 CNG*	1.29 (0.82-2.03)	0.28
Gender	0.82 (0.52-1.30)	0.39
Age^1^	1.52 (1.16-1.98)	0.002
WHO grade^2^	1.92 (1.47-2.48)	0.001
KPS score^3^	1.38 (0.86-2.21)	0.18
Recurrence	44.86 (6.16-327.00)	<0.001
Radiotherapy	0.47 (0.30-0.75)	<0.001
Chemotherapy	1.45 (0.92-2.30)	0.11
Smoking	1.55 (0.96-2.50)	0.07
Drinking	0.84 (0.31-2.30)	0.73
Epilepsy	0.41 (0.25-0.66)	<0.001
*IDH1* mutations	0.30 (0.17-0.53)	<0.001

^*^ HR: hazard ratio with 95% confidence interval (CI); ^1^Age (per 20 years); ^2^WHO grade (I, II, III and IV); ^3^KPS (>80; ≤80). The cases < 4 copies were used as reference.

**Table 5:** Prognostic value of clinicopathological factors and CNG of members of ErbB family using multivariate Cox regression analysis

Characteristics	*EGFR*		*HER2*		*HER3*		*HER4*	
	HR^*^ (95% CI)	*P*	HR^*^ (95% CI)	*P*	HR^*^ (95% CI)	*P*	HR^*^ (95% CI)	*P*
Copy number								
≤4	1.00 (reference)		1.00 (reference)		1.00 (reference)		1.00 (reference)	
>4	3.08 (1.82-5.19)	<0.001	1.66 (0.97-2.85)	0.06	1.71 (1.06-2.76)	0.03	1.76 (1.08-2.87)	0.02
Ages								
20/years	1.35 (1.02-1.78)	0.03	1.60 (1.22-2.10)	<0.001	1.52 (1.15-1.99)	0.003	1.54 (1.18-2.00)	0.002
WHO grade								
I	1.00 (reference)		1.00 (reference)		1.00 (reference)		1.00 (reference)	
II	2.44 (1.04-5.74)	0.04	1.37 (0.60-3.10)	0.45	1.77 (0.77-4.04)	0.18	1.80 (0.78-4.12)	0.17
III	7.69 (3.14-18.88)	<0.001	4.60 (1.98-10.73)	<0.001	5.60 (2.34-13.39)	<0.001	5.43 (2.29-12.90)	<0.001
IV	5.98 (2.37-15.11)	<0.001	3.89 (1.53-9.90)	0.004	5.93 (2.34-15.06)	<0.001	6.74 (2.57-17.72)	<0.001
Radiotherapy								
No	1.00 (reference)		1.00 (reference)		1.00 (reference)		1.00 (reference)	
Yes	0.40 (0.25-0.66)	<0.001	0.50 (0.31-0.80)	0.004	0.51 (0.32-0.81)	0.004	0.51 (0.32-0.81)	0.004
Epilepsy								
No	1.00 (reference)		1.00 (reference)		1.00 (reference)		1.00 (reference)	
Yes	0.44 (0.26-0.74)	0.002	0.40 (0.24-0.68)	<0.001	0.43 (0.25-0.72)	<0.001	0.40 (0.24-0.67)	<0.001

^*^ HR: hazard ratio with 95% confidence interval (CI).

### Association of CNG of members of ErbB family with poor patient survival

Univariate survival analysis was conducted to assess the potential associations of CNG of members of ErbB family with patient survival. As shown in Table 4, *EGFR* CNG (HR =2.31, 95% CI =1.44-3.69, *P* =0.001), *IDH1* mutations (HR =0.3, 95% CI =0.17-0.53, *P* <0.001), increasing age (HR =1.52, 95% CI =1.16-1.98, *P* =0.002), advanced tumor stage (HR =1.92, 95% CI =1.47-2.48, *P* <0.001) and tumor recurrence (HR =44.86, 95% CI =6.16-327.00, *P* <0.001) were significantly correlated with poor survival, while the patients receiving radiotherapy (HR =0.47, 95% CI=0.30-0.75, *P* <0.001) and the patients with epilepsy (HR =0.41, 95% CI=0.25-0.66, *P* <0.001) were more likely to have a better prognosis. To further determine clinical value of CNG of members of ErbB family in predicting patient survival, we conducted multivariate Cox regression analysis. As shown in Table 5, CNG of *EGFR* (HR =3.08, 95% CI =1.82-5.19, *P* <0.001), *HER3* (HR =1.71, 95% CI =1.06-2.76, *P* =0.03) and *HER4* (HR =1.76, 95% CI =1.08-2.87, *P* =0.02) was identified as independent predictors of poor patient survival with respect to ages, WHO grade, radiotherapy and epilepsy.

**Figure 2:**
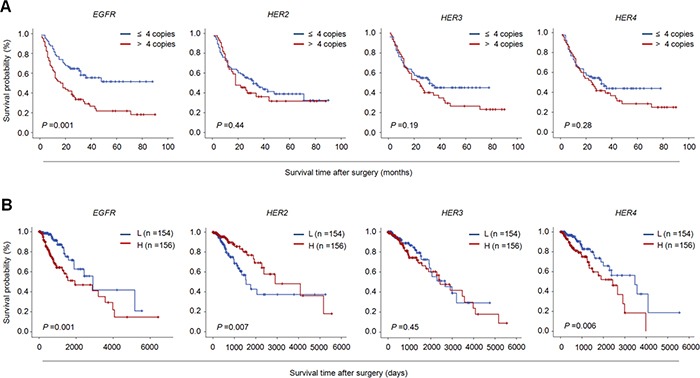
The effect of CNG of members of ErbB family on the survival of glioma patients Kaplan-Meier survival curves were grouped based on the status of CNG of the indicated gene in gliomas from our cohort **(A)** and TCGA cohort **(B)**.“L” and “H” represent low- and high-copy number of the indicated gene, respectively. A median copy number of each indicated gene is used as cut-off value.

Next, we conducted the Kaplan-Meier survival analysis to further validate the effect of CNG of the above genes on survival. As expected, there was a significantly poorer survival in the patients with *EGFR* CNG than those without *EGFR* CNG (18.0 months *vs*. 36.0 months; *P* =0.001) (Figure 2A). This was supported by the TCGA dataset that high copy number of *EGFR* was strongly related with worse patient survival as compared to low copy number (*P* =0.001) (Figure 2B). In addition, the TCGA dataset also showed that high copy number of *HER2* (*P* =0.007) and *HER4* (*P* =0.006) was significantly associated with poor patient survival (Figure 2); however, we did not find significant relationships between CNG of *HER2* and *HER4* and poor patient survival. One possibility is a limited number of glioma patients included in the present study.

**Figure 3:**
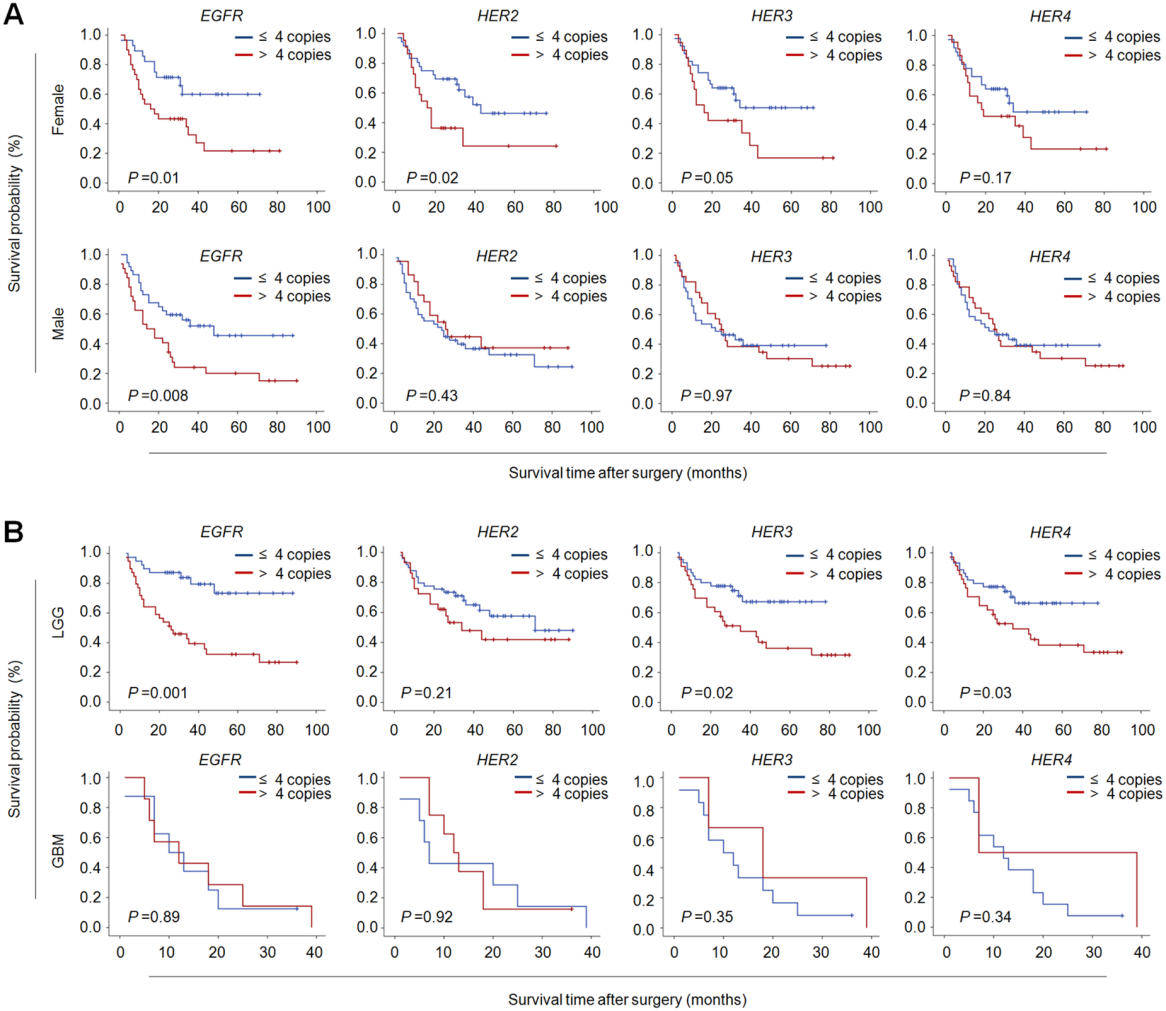
The associations of CNG of members of ErbB family with poor survival of female and low-grade glioma patients Kaplan-Meier survival curves were grouped similar to that shown in Figure 2. **(A)** CNG of members of ErbB family particularly *EGFR*, *HER2* and *HER3* dramatically shortened median survival times in female patients, but not male patients except for *EGFR*. **(B)** CNG of the indicated genes particularly *EGFR*, *HER3* and *HER4* significantly shortened median survival times in LCCs, but not in GBMs.

**Figure 4:**
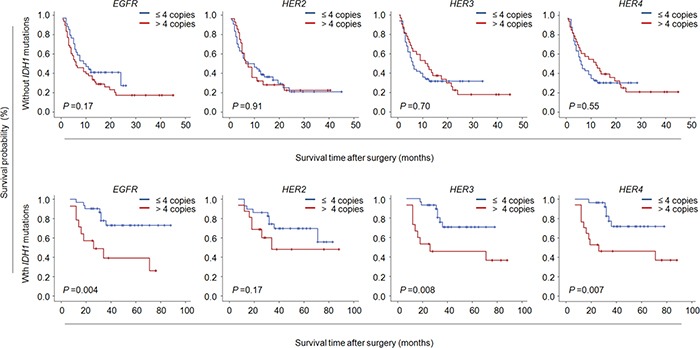
Association of CNG of ErbB family members with poor survival of glioma patients with *IDH1* mutations Kaplan-Meier survival curves were grouped based on the status of *IDH1* mutations. CNG of members of ErbB family dramatically shortened median survival times in the patients with *IDH1* mutations except for *HER2*, while no effect on the survival of the patients without *IDH1* mutations.

Further stratified analysis revealed that CNG of members of ErbB family almost did not influence median survival times of male patients except for *EGFR* (28.3 months *vs*. 50.4 months, *P* =0.008); however, CNG of *EGFR* (31.1 months *vs*. 49.6 months, *P* =0.01), *HER2* (30.8 months *vs*. 46.6 months, *P* =0.02) and *HER3* (29.3 months *vs*. 44.1 months, *P* =0.05) dramatically shortened median survival times in female patients (Figure 3A and Table 6). It is well known that tumor grade significantly affects patient survival, which was supported by our data that tumor grade was positively associated with poor patient survival (Table 5). Thus, the data were stratified according to tumor grade, and prognostic value of CNG of these genes was further evaluated in these patients. As shown in Figure 3B and Table 6, we found that CNG of the above genes had very little effect on the survival of the patients with GBM, while CNG of *EGFR* (40.2 months *vs*. 71.4 months, *P* =0.001), *HER3* (44.8 months *vs*. 58.2 months, *P* =0.02) and *HER4* (46.2 months *vs*. 57.7 months, *P* =0.03) significantly shortened median survival times of the patients with LGG. In addition, given that *IDH1* mutation lead to a better prognosis of glioma patients, we next evaluated the effect of CNG of these genes on the survival of the patients with different *IDH1* mutation status. The results showed that CNG of *EGFR* (40.2 months *vs*. 71.5 months, *P* =0.004), *HER3* (46.7 months *vs*. 63.9 months, *P* =0.008) and *HER4* (47.4 months *vs*. 64.8 months, *P* =0.007) significantly shortened median survival times of the patients with *IDH1* mutation, but no effect on the survival of those without *IDH1* mutations (Figure 4 and Supplementary Table 6).

**Table 6:** The median survival times by grouping with CNG of members of ErbB family

Copy number	Overall		Female		Male		LGG (I/II)		GBM (IV)		Radiotherapy		Non-Radiotherapy	
	Median, months (95% CI)	*P*	Median, months (95% CI)	*P*	Median, months (95% CI)	*P*	Median, months (95% CI)	*P*	Median, months (95% CI)	*P*	Median, months (95% CI)	*P*	Median, months (95% CI)	*P*
≤4	54.4 (45.1-63.8)	0.001	49.6 (39.0-60.2)	0.01	50.4 (38.1-62.8)	0.008	71.4 (61.2-81.5)	0.001	10.0 (1.7-18.3)	0.89	67.0 (56.6-78.4)	0.001	30.3 (21.2-39.5)	0.02
>4	31.0 (22.7-39.2)		31.1 (20.1-42.2)		28.3 (17.5-39.1)		40.2 (29.1-51.3)		12.0 (0.0-24.8)		36.0 (26.0-46.1)		18.1 (6.8-29.4)	
≤4	44.4 (36.0-52.8)	0.43	46.6 (36.1-57.1)	0.02	38.0 (27.5-48.6)	0.40	59.1 (48.5-69.7)	0.21	7.0 (4.4-9.6)	0.92	60.0 (46.2-67.7)	0.04	25.6 (16.3-34.9)	0.54
>4	38.7 (28.0-49.7)		30.8 (16.5-45.1)		44.2 (29.0-59.4)		47.9 (34.1-61.7)		12.0 (7.8-16.2)		40.0 (27.4-52.8)		34.7 (16.9-52.6)	
≤4	42.9 (35.4-50.4)	0.18	44.1 (34.6-53.7)	0.05	38.1 (27.7-48.4)	0.97	58.2 (49.2-67.2)	0.02	10.0 (1.5-18.5)	0.35	52.1 (42.7-61.5)	0.06	25.7 (17.0-34.5)	0.59
>4	36.9 (27.3-46.6)		29.3 (16.5-42.1)		39.6 (27.2-52.1)		44.8 (32.7-56.9)		18.0 (0.4-35.6)		41.5 (30.0-53.1)		25.4 (11.2-39.6)	
≤4	42.4 (34.7-50.0)	0.27	42.9 (32.7-53.1)	0.18	38.5 (28.3-48.7)	0.84	57.7 (48.4-66.9)	0.03	13.7 (8.7-18.7)	0.34	52.6 (43.1-62.1)	0.05	23.9 (15.2-32.6)	0.97
>4	38.2 (28.6-47.7)		33.6 (20.6-46.5)		39.0 (26.4-51.7)		46.2 (34.2-58.1)		23.0 (0.0-54.4)		41.8 (30.3-53.2)		28.4 (13.8-42.9)	

### Impact of CNG of members of ErbB family on radiotherapy outcome of glioma patients

**Figure 5:**
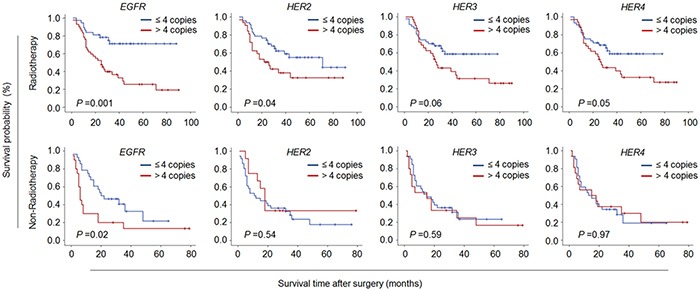
The associations of CNG of members of ErbB family with radiotherapy resistance in glioma patients Kaplan-Meier survival curves were grouped similar to that shown in Figure 2. CNG of members of ErbB family dramatically shortened median survival times in the patients receiving radiotherapy, but not in those who did not receive radiotherapy except for *EGFR*.

It is the fact that postoperative adjuvant radiotherapy may improve survival after surgical resection for glioma. Indeed, our data showed that radiotherapy significantly lengthened median survival of glioma patients (Table 5). Thus, in the present study, we further evaluated the prognostic value of CNG of members of ErbB family in the patients receiving radiotherapy. As shown in Figure 5 and Table 6, CNG of these genes dramatically shortened median survival times in the patients receiving radiotherapy (*EGFR*: 36.0 months *vs*. 67.0 months, *P* =0.001; *HER2*: 40.0 months *vs*. 60.0 months, *P* =0.04; *HER3*: 41.5 months *vs*. 52.1 months, *P* =0.06; *HER4*: 41.8 months *vs*. 52.6 months, *P* =0.05), but not in those who did not receive radiotherapy except for *EGFR* (18.1 months *vs*. 30.3 months, *P* =0.02). In addition, we did not find significant effect of CNG of these genes on chemotherapy in glioma patients (data not shown). Taken together, our data suggest that CNG of *HER2-4* genes may be used as predictors of radiotherapy resistance in gliomas.

## DISCUSSION

Gliomas are the most common and the deadliest brain malignancies all over the world. In general, the progression of glioma is a long-term process with accumulation of a large number of genetic and epigenetic alterations. Genomic amplification, a common mechanism for oncogene overexpression, is one of the most frequent genomic alterations found in human cancers including gliomas [20–27]. It is well-documented that the ErbB family of RTKs has a critical role in the tumorigenesis of many types of cancer [14]. Its members including *EGFR, HER2, HER3* and *HER4* have been reported to be frequently amplified in different types of cancer including gliomas [28, 29]. However, their clinical significance in the prognostic evaluation of glioma patients remains largely unclear.

In the present study, we measured copy number of members of ErbB family (including *EGFR, HER2, HER3* and *HER4*) in a cohort of glioma patients and control subjects using real-time qPCR approach and determined their prognostic significance in gliomas. Our data showed that these genes were frequently amplified in gliomas, but not in control subjects. This was consistent with a previous study that *EGFR* amplification was frequently found in gliomas and could be used as a biomarker for the diagnosis of this cancer [30]. These observations suggest that CNG of members of ErbB family is more likely involved in glioma tumorigenesis. To further explore the associations of these aberrant genetic events with clinicopathological characteristics and clinical outcomes of glioma patients, we defined a copy number of ≥4 as CNG (or gene amplification), the patients were then categorized into two groups: CNG- and Non-CNG-groups. The results demonstrated that CNG of *EGFR, HER3* and *HER4* significantly increased the risk of cancer-related death in glioma patients. This was supported by the findings in some other cancers. For example, CNGs of *EGFR*, *HER2*, *HER3*, and *HER4* had strongly associations with poor overall survival in lung adenocarcinoma with EGFR-activating mutations [31]. In addition, our data also showed that *EGFR*, *HER3* and *HER4* genes were more likely to be amplified in the patients with early-stage tumors, suggesting that these aberrations may be early-stage genetic events, contributing to tumor initiation and tumorigenesis. Interestingly, we found that CNG of *EGFR* and *HER2* was more frequently found in the patients receiving postoperative radiotherapy as compared to those who did not receiving radiotherapy, whereas CNG of *HER3* and *HER4* was more frequently found in the patients who did not receive postoperative chemotherapy relative to those receiving chemotherapy. These data suggest that CNG of *EGFR* and *HER2* may be involved in radiotherapy resistance in gliomas, while CNG of *HER3* and *HER4* may be used as predictors for the sensitivity of glioma cells to chemotherapy. Notably, our data also showed distinct roles of CNG of these genes in female and male patients. However, these findings need to be further validated and elucidated.

Next, we attempted to test the effect of CNG of these genes on clinical outcomes of glioma patients. As expected, our data and the TCGA database showed that CNG of some of these genes was significantly associated with poor patient survival particularly *EGFR*. By using stratified analysis, we surprisingly found that CNG of *EGFR*, *HER2* and *HER3* remarkably shortened median survival times in female patients, but almost did not influence the survival of male patients except for *EGFR*. Similarly, these findings also need to be further confirmed and elucidated. In addition, our data also showed that CNG of *EGFR*, *HER3* and *HER4* significantly shortened median survival times of the patients with early-stage tumors, but had very little effect on the survival of the patients with late-stage tumors, further supporting that these abnormalities may be early genetic events in glioma tumorigenesis. However, a previous study has demonstrated mutation rate of *EGFR* in lung cancer differs between races [32]. Given that the patients investigated in our cohort and TCGA cohort are from different races, thus we speculate that different race backgrounds may lead to some different correlations between CNGs of ErbB family members and clinicopathological characteristics in glioma patients.

It is well known that radiotherapy is a part of multidisciplinary management of several cancers including gliomas. Moreover, there is increasing evidence showing that combined treatment of chemotherapy and radiotherapy is more effective in the care of gliomas as compared to radiotherapy alone [33, 34]. In recent years, several clinical trials of molecularly targeted cancer therapies in glioma have been conducted based on the known functions of the receptors of ErbB family [35, 36]. The two major classes of anti-ErbB therapeutics are monoclonal antibodies and small molecule tyrosine kinase inhibitors (TKI) [37]. AC480 is a highly selective and potent small-molecule inhibitor of EGFR/HER kinase family, and inhibit cancer cell proliferation through targeting EGFR and HER2 kinases [38]. Moreover, AC480 has been demonstrated to enhance radiosensitivity and radioresponse of head and neck squamous cell carcinoma cells *in vitro* and *in vivo* [39]. Notably, we found that CNG of members of ErbB family significantly impacted radiotherapy outcome of glioma patients. CNG of these genes significantly shortened median survival times in the patients receiving radiotherapy. However, the underlying mechanism is still unclear. There is evidence showing that the PI3K/AKT/mTOR cascade has been considered as the predominant downstream pathway of ErbB kinases, promoting radioresistance in various types of cancer including glioma [40–44]. Therefore, we suppose that CNG of members of ErbB family in gliomas induces radiotherapy resistance through activating the PI3K/AKT pathway. Taken together, our data suggest that the combination of TKIs and radiotherapy may be a eutherapeutic strategy for glioma patients with CNG of members of ErbB family, which is strongly supported by several previous studies [45–48].

In summary, our data found frequent CNG of members of ErbB family in patients, and demonstrated that these abnormalities were significantly associated with poor clinical outcomes of glioma patients, particularly in female or low-grade glioma patients. To our knowledge, the present study for the first time demonstrates that CNG of members of ErbB family may contribute to radiotherapy resistance in glioma patients. Altogether, our findings indicate that the above abnormalities may be a trigger of glioma tumorigenesis, and may be used as potentially prognostic markers for glioma patients. In addition, our data also suggest that a combination of anti-ErbB therapeutics and radiotherapy may be an effective strategy for the treatment of glioma patients with CNG or overactivation of members of ErbB family.

## MATERIALS AND METHODS

### Patients and tissue samples

With the approval of our institutional review board and human ethics committee, a total of 127 glioma patients and 16 benign meningiomas as control subjects, who underwent surgery for brain tumors at the Department of Neurosurgery of First Affiliated Hospital of Xi’an Jiaotong University from 2006 to 2012, were randomly enrolled in this study (Supplementary Table 1). None of these patients receive radiotherapy or chemotherapy prior to surgery. Glioma patients received adjuvant radiotherapy and/or chemotherapy after surgery according to standard clinical protocols. All samples were histopathologically classified according to the WHO classification criteria. Overall survival was calculated as time duration starting from surgery until cancer-related death or last follow-up. Clinicopathological data were obtained from the patients’ files or by interview with the patients or their relatives, and were summarized in Table 1. All patients were enrolled after providing a written informed consent.

### DNA preparation

Formalin-fixed paraffin-embedded tissues from each sample were cut at 5 mm, and all tissue sections were reviewed by board-certified pathologists to ensure containing more than 50% tumor cells. DNA was then extracted according to a previously described protocol [49]. In brief, the sections were first treated with xylene for 12 h at room temperature for deparaffinization, followed by digestion with 1% sodium dodecylsulfate (SDS) and proteinase K at 48°C for 48h. Genomic DNA was then isolated from these tissues using a standard protocol.

### Copy number analysis

Copy number of members of ErbB family was analyzed in gliomas and control subjects by a well-established real-time qPCR approach, which was previously validated by fluorescence in situ hybridization (FISH) [50]. Primer Express 3.0 software (Applied Biosystems, Foster City, CA) was utilized to design specific PCR primers and TaqMan probes for the amplification of the indicated genes and internal control *β-actin*. TaqMan probes were labeled at the 5′ end with a fluorescent reporter 6-carboxyfluorescein (6FAM) and at the 3′ end with a fluorescent quencher 6-carboxy-tetramethylrhodamine (TAMRA). The sequences were presented in Supplementary Table 7. The PCR reaction was performed according to a previously described protocol [50, 51]. Each sample was run in triplicate, and *β-actin* was performed in parallel to normalize the input DNA. Serially diluted leukocyte DNA was used to establish standard curves. Copy number was calculated as previously described. A copy number ≥4 was defined as gene amplification (or copy number gain).

### Detection of *IDH1* mutations

A fragment of 129 bp length spanning the catalytic domain of *IDH1* including codon 132 was amplified by PCR with the following primers: 5′-CGG TCT TCA GAG AAG CCA TT-3′ (forward) and 5′-GCA AAA TCA CAT TAT TGC CAA C-3′ (reverse). PCR products were analyzed by Sanger sequencing.

### Statistical analysis

Statistical analysis was performed using the SPSS 15.0 software (Chicago, IL, USA). *P* value <0.05 was considered statistically significant. Mann-Whitney *U*-test was applied to compare copy number of the indicated genes between gliomas and control subjects. SPSS 15.0 software was used for the univariately logistic regression analysis of the association of CNG of the indicated genes with clinicopathological features of giloma patients. Multivariate analysis was performed to calculate multivariable-adjusted odds ratios (ORs) and 95% confidence intervals (CIs) for CNG of the indicated genes, and other factors such as WHO grade, recurrence, radiotherapy, chemotherapy and survival status. Cancer-related survival was calculated from the date of the operation to cancer-related death or last follow-up. Kaplan-Meier survival analysis was performed to evaluate the impact of CNG of the indicated genes on patient survival. Log-rank test was used to analyze the differences between curves. The effect of CNG of the indicated genes on the independent survival of age, WHO grade, radiotherapy and epilepsy was determined by multivariate Cox regression analysis.

## SUPPLEMENTARY MATERIALS FIGURES AND TABLES




