# Supplementary material for: Genomic copy number gains of ErbB family members predict poor clinical outcomes in glioma patients
Source: Oncotarget. 2017 Sep 23;8(54):92275–88. doi: 10.18632/oncotarget.21228 (PMC5696180; doi:10.18632/oncotarget.21228)
Supplement: Supplementary file 1 [file oncotarget-08-92275-s001.pdf]

Genomic copy number gains of ErbB family members predict poor clinical outcomes in glioma patients

SUPPLEMENTARY MATERIALS

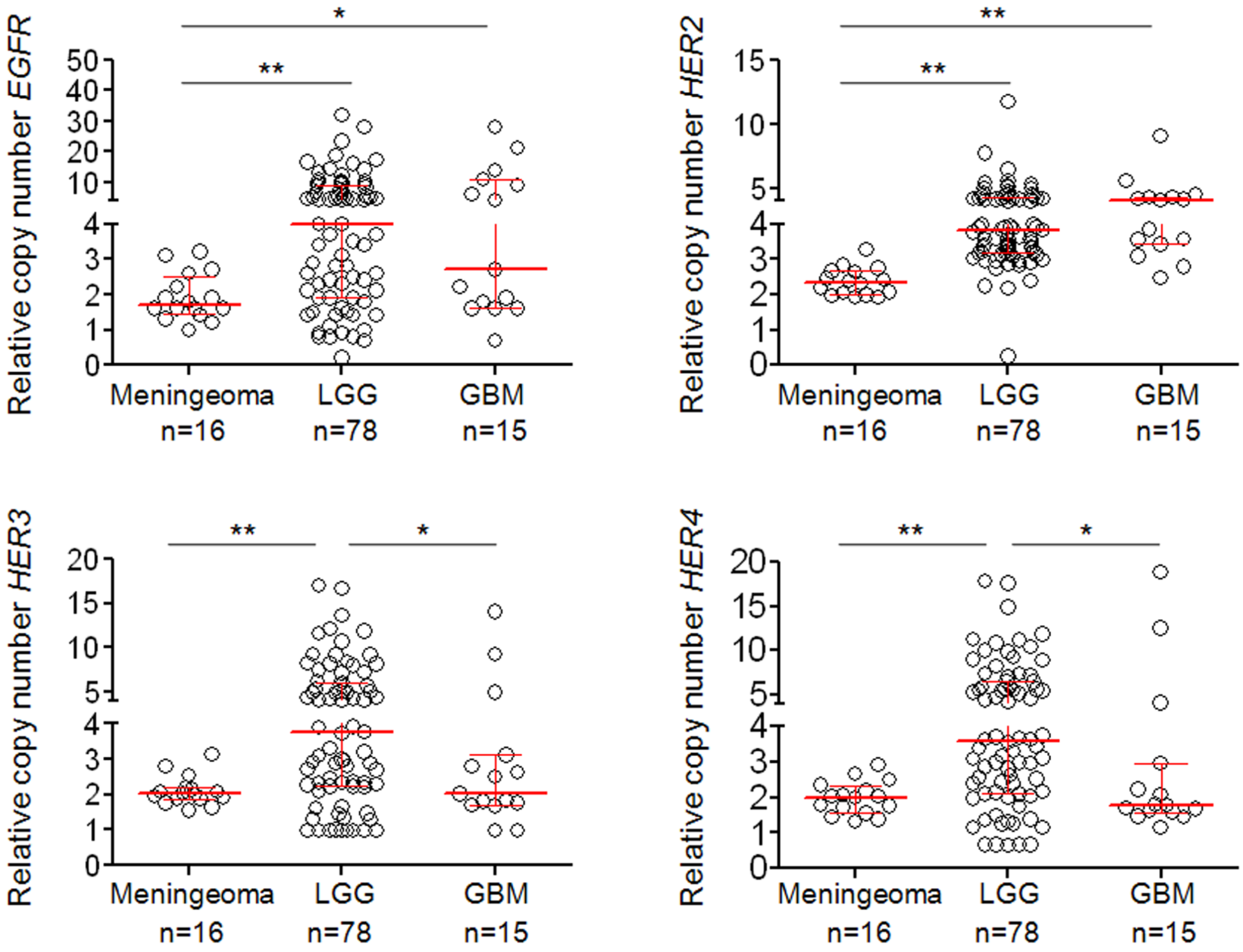

Supplementary Figure 1: Compare the copy number of ErbB family members in the different grades of gliomas and control subjects. Copy number of *EGFR*, *HER2*, *HER3* and *HER4* of each case was determined by a qPCR assay. Each circle represents copy number of the indicated gene of an individual case. Horizontal lines indicate median and interquartile range. T: tumor tissues; N: control subjects.

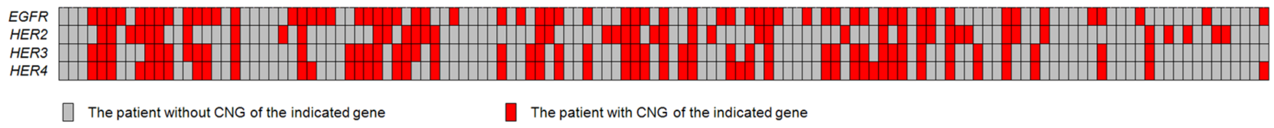

Supplementary Figure 2: CNG of ErbB family members in 127 glioma patients. Red box represents the patient with CNG of the indicated gene.

Supplementary Table 1: Copy number of members of ErbB family and clinicopathological characteristics in 127 glioma patients

CN: copy number.

See Supplementary File 1

Supplementary Table 2: CNG of members of ErbB family in female patients: univariate associations with clinicopathological characteristics

Characteristics	*EGFR* CNG		*HER2* CNG		*HER3* CNG		*HER4* CNG	
	OR* (95% CI)	*P*	OR* (95% CI)	*P*	OR* (95% CI)	*P*	OR* (95% CI)	*P*
Age[1]	0.98 (0.55-1.74)	0.94	0.75 (0.41-1.37)	0.35	0.84 (0.46-1.56)	0.59	0.83 (0.46-1.50)	0.53
WHO grade[2]	0.65 (0.33-1.28)	0.21	0.84 (0.43-1.67)	0.62	0.72 (0.35-1.48)	0.37	0.84 (0.43-1.67)	0.62
KPS score[3]	0.76 (0.27-2.12)	0.60	0.55 (0.19-1.62)	0.28	0.86 (0.29-2.56)	0.78	1.00 (0.35-2.89)	1.00
Recurrence	3.47 (1.08-11.08)	0.03	3.21 (0.90-11.44)	0.07	3.71 (0.93-14.87)	0.06	2.16 (0.65-7.19)	0.21
Radiotherapy	3.00 (0.93-9.63)	0.06	1.33 (0.42-4.28)	0.63	1.40 (0.41-4.74)	0.59	0.94 (0.30-2.96)	0.92
Chemotherapy	1.04 (0.36-3.04)	0.94	1.01 (0.34-3.05)	0.99	1.46 (0.47-4.49)	0.52	0.73 (0.24-2.25)	0.59
Survival status[4]	4.20 (1.40-12.60)	0.01	2.68 (1.08-8.15)	0.04	3.62 (1.09-12.05)	0.04	2.68 (1.08-8.15)	0.04
Epilepsy	0.66 (0.24-1.87)	0.44	0.33 (0.11-1.02)	0.06	0.23 (0.16-1.54)	0.44	0.83 (0.29-2.41)	0.74

*OR: odds ratio with 95% confidence interval; [1]Age (per 20 years); [2]WHO grade (I, II, III and IV); [3]KPS (>80; ≤80); [4]Survival status (alive *vs*. dead). The cases < 4 copies were used as reference.

Supplementary Table 3: CNG of members of ErbB family in male patients: univariate associations with clinicopathological characteristics

Characteristics	*EGFR* CNG		*HER2* CNG		*HER3* CNG		*HER4* CNG	
	OR* (95% CI)	*P*	OR* (95% CI)	*P*	OR* (95% CI)	*P*	OR* (95% CI)	*P*
Age[1]	1.58 (0.91-2.74)	0.11	0.48 (0.26-0.89)	0.02	1.06 (0.62-1.81)	0.84	1.14 (0.67-1.95)	0.64
WHO grade[2]	1.01 (0.59-1.69)	0.98	1.32 (0.75-2.32)	0.33	0.72 (0.35-1.48)	0.37	0.48 (0.27-0.88)	0.02
KPS score[3]	1.38 (0.49-3.86)	0.53	0.55 (0.19-1.60)	0.27	1.29 (0.46-3.68)	0.63	1.73 (0.59-5.02)	0.31
Recurrence	3.11 (0.76-12.68)	0.11	0.59 (0.17-2.14)	0.43	1.46 (0.39-5.39)	0.58	1.46 (0.39-5.39)	0.58
Radiotherapy	0.98 (0.37-2.54)	0.97	2.78 (0.93-8.35)	0.07	1.71 (0.64-4.59)	0.28	2.22 (0.81-6.04)	0.12
Chemotherapy	0.63 (0.24-1.66)	0.35	1.13 (0.41-3.12)	0.82	0.35 (0.12-0.96)	0.04	0.35 (0.12-0.96)	0.04
Survival status[4]	4.57 (1.53-13.70)	0.007	0.75 (0.26-2.11)	0.58	1.77 (0.63-4.95)	0.28	1.77 (0.63-4.95)	0.28
Smoking	1.51 (0.58-3.92)	0.39	0.42 (0.15-1.20)	0.11	1.98 (0.74-5.25)	0.17	1.54 (0.59-4.06)	0.38
Drinking	0.21 (0.02-1.87)	0.16	0.40 (0.04-3.65)	0.42	0.71 (0.12-4.18)	0.71	0.27 (0.03-2.42)	0.24
Epilepsy	0.55 (0.21-1.46)	0.23	1.61 (0.58-4.48)	0.36	1.06 (0.40-2.80)	0.91	1.06 (0.40-2.80)	0.91

*OR: odds ratio with 95% confidence interval; [1]Age (per 20 years); [2]WHO grade (I, II, III and IV); [3]KPS (>80; ≤80); [4]Survival status (alive *vs*. dead). The cases < 4 copies were used as reference.

Supplementary Table 4: CNG of members of ErbB family in female patients: multivariate models assessing WHO grade, recurrence, radiotherapy, chemotherapy and survival status

Characteristics	*EGFR* CNG		*HER2* CNG		*HER3* CNG		*HER4* CNG	
	OR* (95% CI)	*P*	OR* (95% CI)	*P*	OR* (95% CI)	*P*	OR* (95% CI)	*P*
WHO grade[1]	0.34 (0.13-0.88)	0.03	0.61 (0.29-1.31)	0.21	0.51 (0.23-1.14)	0.09	0.62 (0.29-1.34)	0.22
Recurrence	2.19 (0.33-14.42)	0.99	2.87 (0.44-18.57)	0.27	2.45 (0.31-19.61)	0.39	1.20 (0.17-8.46)	0.86
Radiotherapy	3.40 (0.86-13.47)	0.08	1.21 (0.34-4.27)	0.76	1.16 (0.31-4.40)	0.83	0.89 (0.26-3.07)	0.86
Chemotherapy	0.40 (0.09-1.71)	0.22	0.65 (0.19-2.25)	0.49	0.89 (0.25-3.24)	0.87	0.47 (0.13-1.67)	0.24
Survival status[2]	6.76 (1.06-42.99)	0.04	1.88 (0.36-9.86)	0.45	2.82 (0.46-17.26)	0.26	3.76 (0.60-23.58)	0.16

*OR: odds ratio with 95% confidence interval (CI); [1]WHO grade (I, II, III and IV); [2]Survival status (alive *vs*. dead). The cases < 4 copies were used as reference.

Supplementary Table 5: CNG of members of ErbB family in male patients: multivariate models assessing WHO grade, recurrence, radiotherapy, chemotherapy and survival status

Characteristics	*EGFR* CNG		*HER2* CNG		*HER3* CNG		*HER4* CNG	
	OR* (95% CI)	*P*	OR* (95% CI)	*P*	OR* (95% CI)	*P*	OR* (95% CI)	*P*
WHO grade[1]	0.71 (0.37-1.35)	0.29	1.69 (0.88-3.24)	0.12	0.33 (0.15-0.73)	0.006	0.26 (0.11-0.64)	0.003
Recurrence	0.78 (0.12-5.23)	0.79	0.44 (0.08-2.52)	0.35	0.75 (0.11-5.08)	0.77	0.66 (0.09-4.86)	0.68
Radiotherapy	2.43 (0.69-8.50)	0.17	3.91 (1.08-14.11)	0.04	4.02 (1.00-16.23)	0.04	7.28 (1.56-33.98)	0.01
Chemotherapy	0.48 (0.16-1.46)	0.19	1.03 (0.34-3.10)	0.96	0.17 (0.04-0.64)	0.009	0.12 (0.03-0.53)	0.005
Survival status[2]	10.68 (1.91-59.78)	0.007	1.32 (0.28-6.21)	0.73	9.41 (1.51-58.50)	0.006	16.00 (2.22-115.65)	0.006

*OR: odds ratio with 95% confidence interval (CI); [1]WHO grade (I, II, III and IV); [2]Survival status (alive *vs*. dead). The cases < 4 copies were used as reference.

Supplementary Table 6: The effect of CNG of ErbB family members on median survival times of glioma patients grouping by *IDH1* mutations

Genes/Copy number	Without *IDH1* mutations		With *IDH1* mutations	
	Median, months(95% CI)	*P*	Median, months(95% CI)	*P*
EGFR				
≤4	18.0 (7.7-28.3)	0.17	71.5 (61.0-82.1)	0.004
>4	12.0 (4.4-19.6)		40.2 (24.8-55.6)	
HER2				
≤4	15.0 (1.8-28.2)	0.91	63.5 (53.1-74.0)	0.17
>4	13.0 (7.1-18.9)		53.1 (34.1-72.1)	
HER3				
≤4	12.0 (8.5-15.5)	0.70	63.9 (55.0-72.8)	0.008
>4	22.0 (10.9-33.1)		46.7 (28.6-64.9)	
HER4				
≤4	12.0 (8.6-15.4)	0.55	64.8 (55.8-73.9)	0.007
>4	24.0 (10.5-37.5)		47.4 (30.4-64.3)	

Supplementary Table 7: The primer and TaqMan probe sequences used in this study

Genes	Forward primer sequence (5'→3')	Probe sequence (5'→3')	Reverse primer sequence (5'→3')	Amplification efficiency(%)
EGFR	CTGCAGATCATCAGAGGAAATATG	6FAM-CGAAAATTCCTATGCCTTAG-TAMRA	CAGTCCGGTTTTATTTGCATCATAG	93.6
HER2	ACCATCCTGCCTCTCCTTCC	6FAM-CATCGTCCTCCAGCAGTGAGCGGT-TAMRA	TCCTCAGCATCCACCAGGTC	97.2
HER3	CTACGGAAGAGCGACTAGACATC	6FAM-AGCAGACCCTTTCCCTCACCGCAG-TAMRA	TCCCTGACTCCCTATTCTCACC	91.1
HER4	CCCTGAAGCCAGGCACTGT	6FAM-CTGCCGCCTCCACCTTACAGACACC-TAMRA	CCTAAAAAACCACAACTGAGCTTACA	95.4
β-actin	TCACCCACACTGTGCCCATCTACGA	6FAM-ATGCCCTCCCCCATGCCATCC-TAMRA	TCGGTGAGGATCTTCATGAGGTA	95.7
