# Supplementary material for: Genomic copy number gains of ErbB family members predict poor clinical outcomes in glioma patients
Source: Oncotarget. 2017 Sep 23;8(54):92275–88. doi: 10.18632/oncotarget.21228 (PMC5696180; doi:10.18632/oncotarget.21228)
Supplement: Supplementary file 2 [file oncotarget-08-92275-s002.docx]

**Table S1:** Copy number of members of ErbB family and clinicopathological characteristics in 127 glioma patients

| No. | Age  (per 20y) | Gender | WHO grade | KPS  >70 | Recurrence | Radio-  therapy | Chemo-  therapy | Smoking | Drinking | Epilepsy | Survival (months) | Dead | EGFR  CN | HER2  CN | HER3  CN | HER4  CN |
| --- | --- | --- | --- | --- | --- | --- | --- | --- | --- | --- | --- | --- | --- | --- | --- | --- |
| 1 | 2 | male | 1 | No | Yes | Yes | No | No | No | Yes | 78 | No | 3.7 | 3.9 | 4.0 | 3.6 |
| 2 | 3 | female | 1 | No | No | Yes | No | No | No | No | 50 | No | 0.9 | 3.1 | 1.4 | 1.3 |
| 3 | 2 | male | 1 | Yes | Yes | Yes | Yes | No | No | Yes | 59 | No | 1.1 | 2.9 | 1.6 | 1.3 |
| 4 | 3 | female | 1 | No | No | No | No | No | No | No | 76 | No | 16.7 | 4.0 | 9.2 | 6.4 |
| 5 | 1 | male | 1 | No | No | No | No | No | No | Yes | 79 | No | 10.4 | 4.4 | 8.3 | 10.0 |
| 6 | 3 | female | 2 | No | Yes | Yes | No | No | No | No | 18 | Yes | 28.0 | 4.3 | 5.6 | 5.4 |
| 7 | 3 | female | 2 | No | No | Yes | No | No | No | Yes | 30 | No | 9.6 | 2.2 | 1.0 | 0.7 |
| 8 | 1 | female | 4 | No | Yes | No | No | No | No | No | 13 | Yes | 1.9 | 4.1 | 1.0 | 1.7 |
| 9 | 4 | male | 3 | Yes | Yes | Yes | Yes | No | No | No | 1 | Yes | 5.1 | 4.9 | 3.4 | 4.2 |
| 10 | 3 | male | 4 | Yes | Yes | No | No | No | No | No | 7 | Yes | 28.0 | 9.1 | 14.0 | 18.8 |
| 11 | 3 | female | 3 | Yes | Yes | Yes | Yes | No | No | Yes | 16 | Yes | 5.1 | 4.5 | 4.7 | 4.7 |
| 12 | 4 | female | 3 | No | Yes | No | No | No | No | Yes | 3 | Yes | 6.7 | 3.0 | 4.8 | 5.4 |
| 13 | 4 | male | 3 | Yes | Yes | Yes | No | Yes | No | No | 10 | Yes | 0.9 | 3.3 | 1.4 | 1.2 |
| 14 | 3 | female | 2 | No | Yes | No | Yes | No | No | No | 5 | Yes | 5.2 | 5.0 | 4.1 | 3.7 |
| 15 | 2 | female | 4 | No | Yes | Yes | No | No | No | Yes | 39 | Yes | 13.8 | 3.5 | 9.2 | 12.5 |
| 16 | 3 | male | 2 | No | Yes | Yes | Yes | No | No | Yes | 12 | Yes | 4.6 | 3.8 | 4.3 | 4.6 |
| 17 | 4 | male | 4 | Yes | Yes | No | No | Yes | No | No | 6 | Yes | 11.1 | 3.4 | 2.0 | 1.8 |
| 18 | 2 | male | 2 | Yes | No | No | Yes | Yes | No | Yes | 31 | No | 3.4 | 4.0 | 1.0 | 3.6 |
| 19 | 2 | female | 2 | No | Yes | No | No | No | No | No | 28 | No | 7.6 | 4.5 | 7.1 | 7.4 |
| 20 | 3 | male | 3 | Yes | Yes | No | Yes | Yes | Yes | No | 22 | Yes | 2.3 | 3.3 | 2.2 | 2.2 |
| 21 | 2 | male | 1 | Yes | No | No | Yes | No | No | Yes | 35 | No | 2.1 | 3.9 | 2.2 | 1.7 |
| 22 | 3 | female | 2 | Yes | No | Yes | Yes | No | No | Yes | 49 | No | 2.6 | 3.1 | 2.9 | 3.0 |
| 23 | 3 | female | 2 | No | No | Yes | No | No | No | No | 52 | No | 1.9 | 3.6 | 2.3 | 2.1 |
| 24 | 4 | female | 4 | No | Yes | Yes | Yes | No | No | No | 18 | Yes | 1.6 | 4.1 | 1.8 | 1.5 |
| 25 | 3 | male | 3 | Yes | Yes | No | No | Yes | No | Yes | 6 | Yes | 6.5 | 3.4 | 1.5 | 1.3 |
| 26 | 2 | male | 2 | No | Yes | No | No | No | No | Yes | 18 | Yes | 4.4 | 5.0 | 4.9 | 4.4 |
| 27 | 1 | female | 2 | No | No | Yes | No | No | No | No | 68 | No | 4.4 | 2.8 | 4.0 | 5.0 |
| 28 | 3 | male | 2 | Yes | Yes | No | Yes | No | No | No | 8 | Yes | 4.8 | 3.2 | 2.1 | 2.0 |
| 29 | 3 | male | 3 | Yes | Yes | No | No | Yes | No | No | 4 | Yes | 22.8 | 4.0 | 2.3 | 2.0 |
| 30 | 4 | male | 2 | Yes | No | Yes | No | No | Yes | Yes | 41 | No | 1.4 | 3.6 | 2.9 | 3.2 |
| 31 | 4 | male | 2 | Yes | No | Yes | No | Yes | No | Yes | 83 | No | 2.4 | 3.2 | 7.2 | 9.0 |
| 32 | 3 | female | 3 | Yes | Yes | Yes | Yes | No | No | Yes | 32 | No | 8.5 | 3.3 | 11.1 | 10.0 |
| 33 | 2 | male | 3 | Yes | Yes | Yes | No | Yes | No | No | 14 | Yes | 7.1 | 1.4 | 5.7 | 5.8 |
| 34 | 4 | male | 2 | No | Yes | Yes | Yes | Yes | No | No | 12 | Yes | 16.1 | 5.1 | 13.6 | 14.9 |
| 35 | 1 | female | 2 | No | No | Yes | No | No | No | Yes | 57 | No | 10.2 | 4.5 | 3.3 | 3.1 |
| 36 | 1 | male | 1 | Yes | No | Yes | No | No | No | Yes | 90 | No | 17.4 | 4.0 | 8.1 | 7.0 |
| 37 | 2 | male | 2 | Yes | Yes | Yes | No | No | No | Yes | 46 | No | 2.5 | 4.2 | 16.6 | 17.8 |
| 38 | 3 | male | 4 | No | Yes | No | Yes | Yes | Yes | No | 18 | Yes | 9.1 | 5.6 | 4.9 | 4.0 |
| 39 | 3 | male | 4 | Yes | Yes | Yes | Yes | Yes | No | No | 12 | Yes | 21.3 | 4.3 | 1.7 | 1.5 |
| 40 | 2 | male | 2 | Yes | No | Yes | No | No | No | No | 88 | No | 2.8 | 4.2 | 5.0 | 5.6 |
| 41 | 2 | male | 2 | No | Yes | Yes | Yes | No | No | No | 43 | No | 2.3 | 3.3 | 3.9 | 3.0 |
| 42 | 4 | male | 3 | No | Yes | No | No | No | No | No | 8 | Yes | 5.2 | 2.7 | 3.0 | 2.4 |
| 43 | 4 | female | 2 | No | Yes | Yes | No | No | No | Yes | 71 | No | 3.7 | 3.4 | 2.2 | 2.0 |
| 44 | 3 | male | 3 | Yes | Yes | No | Yes | No | No | No | 6 | Yes | 3.3 | 3.5 | 1.2 | 1.0 |
| 45 | 2 | male | 2 | No | Yes | No | No | No | No | No | 15 | Yes | 1.5 | 3.0 | 1.0 | 2.2 |
| 46 | 3 | female | 4 | Yes | Yes | No | No | No | No | No | 1 | Yes | 0.7 | 2.8 | 1.8 | 1.6 |
| 47 | 2 | male | 1 | Yes | Yes | Yes | No | Yes | No | No | 71 | Yes | 5.5 | 0.2 | 5.2 | 5.5 |
| 48 | 3 | male | 1 | Yes | Yes | Yes | Yes | Yes | No | No | 4 | Yes | 1.4 | 2.9 | 1.5 | 1.1 |
| 49 | 4 | female | 2 | No | Yes | Yes | Yes | No | No | No | 20 | Yes | 5.4 | 3.4 | 1.0 | 0.7 |
| 50 | 3 | male | 2 | Yes | Yes | No | No | Yes | Yes | No | 48 | Yes | 0.8 | 2.9 | 7.4 | 5.7 |
| 51 | 3 | female | 2 | Yes | Yes | Yes | Yes | No | No | No | 10 | Yes | 11.1 | 4.2 | 12.1 | 9.2 |
| 52 | 1 | female | 3 | No | Yes | Yes | No | No | No | No | 6 | Yes | 38.7 | 6.4 | 1.0 | 0.7 |
| 53 | 4 | male | 2 | No | Yes | No | No | Yes | No | No | 5 | Yes | 4.1 | 3.0 | 6.0 | 5.3 |
| 54 | 3 | male | 2 | Yes | Yes | Yes | No | Yes | No | Yes | 56 | No | 2.3 | 3.2 | 2.7 | 2.4 |
| 55 | 2 | female | 2 | Yes | Yes | Yes | Yes | No | No | Yes | 31 | Yes | 2.4 | 3.1 | 3.0 | 2.5 |
| 56 | 4 | female | 2 | Yes | Yes | Yes | Yes | No | No | No | 11 | Yes | 4.7 | 3.2 | 4.8 | 5.9 |
| 57 | 2 | male | 3 | Yes | Yes | No | No | Yes | No | No | 11 | Yes | 1.5 | 3.8 | 2.3 | 2.2 |
| 58 | 1 | male | 4 | Yes | Yes | Yes | Yes | No | No | No | 10 | Yes | 1.8 | 4.2 | 2.6 | 2.1 |
| 59 | 3 | female | 2 | Yes | No | Yes | No | No | No | No | 23 | No | 0.2 | 4.7 | 2.7 | 2.8 |
| 60 | 2 | male | 2 | No | Yes | Yes | Yes | Yes | No | Yes | 22 | Yes | 19.0 | 6.4 | 11.6 | 9.8 |
| 61 | 3 | male | 2 | No | Yes | Yes | No | No | No | Yes | 44 | Yes | 23.5 | 4.7 | 17.0 | 17.5 |
| 62 | 3 | male | 1 | Yes | Yes | Yes | Yes | No | No | No | 59 | No | 7.0 | 3.3 | 5.8 | 7.2 |
| 63 | 2 | female | 2 | Yes | Yes | Yes | No | No | No | Yes | 24 | No | 0.9 | 4.1 | 1.6 | 1.5 |
| 64 | 2 | female | 1 | Yes | Yes | Yes | Yes | No | No | No | 8 | Yes | 10.7 | 5.4 | 10.7 | 11.1 |
| 65 | 1 | male | 3 | No | Yes | Yes | No | No | No | No | 26 | No | 1.7 | 3.2 | 2.3 | 1.4 |
| 66 | 3 | male | 3 | Yes | Yes | Yes | Yes | Yes | No | No | 24 | Yes | 2.6 | 3.8 | 4.7 | 6.2 |
| 67 | 4 | female | 2 | No | Yes | Yes | No | No | No | No | 9 | Yes | 32.0 | 7.7 | 11.8 | 11.8 |
| 68 | 2 | female | 2 | No | No | No | No | No | No | Yes | 55 | No | 1.5 | 3.6 | 1.0 | 1.4 |
| 69 | 2 | male | 2 | No | Yes | Yes | Yes | No | No | Yes | 50 | No | 1.4 | 4.2 | 2.2 | 2.1 |
| 70 | 4 | female | 3 | No | Yes | Yes | No | No | No | Yes | 26 | No | 7.7 | 4.0 | 3.5 | 3.4 |
| 71 | 4 | female | 1 | Yes | Yes | No | No | No | No | No | 35 | Yes | 4.3 | 3.0 | 4.3 | 4.7 |
| 72 | 4 | female | 3 | No | Yes | No | No | No | No | Yes | 19 | Yes | 0.5 | 1.3 | 1.0 | 11.6 |
| 73 | 3 | male | 3 | Yes | Yes | Yes | No | Yes | No | No | 28 | Yes | 13.5 | 3.9 | 7.3 | 7.6 |
| 74 | 2 | male | 2 | Yes | No | Yes | Yes | No | No | Yes | 38 | No | 14.6 | 4.6 | 4.1 | 3.1 |
| 75 | 2 | male | 2 | Yes | Yes | Yes | Yes | No | No | Yes | 26 | Yes | 4.4 | 4.5 | 5.0 | 5.5 |
| 76 | 3 | male | 4 | Yes | Yes | Yes | No | Yes | No | No | 25 | Yes | 6.1 | 3.6 | 1.8 | 1.6 |
| 77 | 1 | male | 4 | No | Yes | No | Yes | No | No | No | 20 | Yes | 1.6 | 3.1 | 3.1 | 3.0 |
| 78 | 3 | female | 4 | Yes | Yes | No | Yes | No | No | Yes | 7 | Yes | 2.7 | 3.8 | 1.0 | 1.1 |
| 79 | 2 | female | 3 | Yes | Yes | Yes | Yes | No | No | Yes | 4 | Yes | 4.6 | 2.6 | 2.3 | 2.0 |
| 80 | 3 | female | 2 | No | No | Yes | No | No | No | Yes | 24 | No | 1.6 | 3.9 | 1.0 | 0.7 |
| 81 | 3 | male | 2 | Yes | Yes | No | No | Yes | No | No | 3 | Yes | 14.2 | 3.3 | 8.0 | 10.9 |
| 82 | 2 | female | 2 | Yes | Yes | Yes | Yes | No | No | No | 7 | Yes | 5.5 | 3.9 | 5.0 | 5.8 |
| 83 | 4 | female | 2 | No | Yes | Yes | Yes | No | No | No | 8 | Yes | 4.0 | 4.2 | 3.7 | 3.3 |
| 84 | 4 | male | 1 | Yes | Yes | Yes | No | Yes | No | No | 25 | Yes | 4.9 | 3.8 | 4.3 | 4.2 |
| 85 | 2 | female | 2 | No | No | Yes | No | No | No | Yes | 31 | No | 6.7 | 3.8 | 6.2 | 7.4 |
| 86 | 2 | female | 3 | Yes | No | No | No | No | No | Yes | 37 | No | 3.6 | 3.5 | 3.8 | 4.5 |
| 87 | 2 | female | 3 | No | Yes | Yes | No | No | No | Yes | 12 | Yes | 23.8 | 4.8 | 11.3 | 11.0 |
| 88 | 3 | male | 3 | Yes | Yes | No | No | Yes | No | No | 2 | Yes | 4.4 | 3.9 | 4.8 | 4.8 |
| 89 | 2 | female | 2 | No | No | Yes | No | No | No | No | 81 | No | 13.2 | 4.1 | 8.2 | 11.2 |
| 90 | 3 | female | 2 | No | Yes | Yes | Yes | No | No | Yes | 31 | No | 2.8 | 3.9 | 2.9 | 3.0 |
| 91 | 2 | male | 1 | Yes | Yes | Yes | Yes | Yes | No | No | 27 | Yes | 12.2 | 5.6 | 8.1 | 6.6 |
| 92 | 3 | female | 2 | Yes | Yes | Yes | No | No | No | Yes | 4 | Yes | 4.0 | 4.3 | 3.2 | 3.4 |
| 93 | 2 | male | 2 | Yes | Yes | No | Yes | No | Yes | Yes | 36 | Yes | 3.1 | 3.3 | 3.9 | 3.7 |
| 94 | 3 | male | 2 | Yes | Yes | Yes | No | Yes | No | Yes | 25 | No | 9.7 | 5.3 | 9.1 | 8.8 |
| 95 | 1 | male | 4 | No | Yes | No | No | No | No | No | 7 | Yes | 2.2 | 4.3 | 2.8 | 1.7 |
| 96 | 3 | female | 3 | No | Yes | No | No | No | No | Yes | 18 | Yes | 3.1 | 4.1 | 3.6 | 3.3 |
| 97 | 3 | male | 3 | Yes | Yes | No | No | Yes | No | No | 4 | Yes | 3.6 | 3.9 | 5.0 | 4.3 |
| 98 | 4 | male | 2 | No | Yes | No | No | Yes | No | No | 12 | Yes | 0.8 | 2.4 | 1.0 | 0.7 |
| 99 | 3 | male | 2 | Yes | No | No | No | No | No | Yes | 27 | No | 1.9 | 3.2 | 2.5 | 2.6 |
| 100 | 3 | male | 2 | Yes | Yes | Yes | No | Yes | No | Yes | 76 | No | 9.1 | 5.2 | 4.9 | 8.2 |
| 101 | 4 | female | 1 | Yes | Yes | Yes | Yes | No | No | No | 10 | Yes | 9.5 | 11.7 | 3.0 | 3.4 |
| 102 | 3 | female | 2 | Yes | No | No | Yes | No | No | No | 32 | No | 3.4 | 3.4 | 2.4 | 2.0 |
| 103 | 2 | female | 3 | Yes | Yes | Yes | Yes | No | No | Yes | 12 | Yes | 2.0 | 3.5 | 4.9 | 4.8 |
| 104 | 3 | female | 2 | Yes | Yes | Yes | No | No | No | Yes | 34 | Yes | 5.3 | 4.4 | 1.0 | 0.7 |
| 105 | 2 | female | 2 | Yes | No | No | Yes | No | No | Yes | 65 | No | 0.7 | 2.9 | 1.3 | 1.1 |
| 106 | 3 | male | 3 | Yes | Yes | No | Yes | No | No | Yes | 5 | Yes | 1.8 | 3.4 | 2.4 | 2.9 |
| 107 | 2 | male | 2 | Yes | Yes | Yes | Yes | No | No | Yes | 31 | No | 0.8 | 3.4 | 1.3 | 1.3 |
| 108 | 2 | male | 2 | No | Yes | Yes | Yes | Yes | No | No | 34 | No | 3.5 | 3.3 | 3.8 | 3.7 |
| 109 | 2 | male | 3 | Yes | Yes | No | No | No | No | No | 1 | Yes | 7.5 | 3.5 | 1.0 | 0.7 |
| 110 | 1 | female | 1 | Yes | Yes | Yes | No | No | No | No | 43 | Yes | 8.3 | 3.6 | 9.2 | 10.4 |
| 111 | 3 | male | 3 | No | Yes | Yes | Yes | Yes | No | Yes | 62 | No | 1.8 | 4.0 | 2.0 | 1.9 |
| 112 | 3 | female | 2 | Yes | No | Yes | No | No | No | Yes | 41 | No | 2.6 | 3.5 | 3.1 | 3.6 |
| 113 | 3 | female | 3 | No | Yes | Yes | No | No | No | Yes | 32 | No | 1.3 | 3.0 | 1.8 | 1.8 |
| 114 | 4 | female | 3 | Yes | Yes | Yes | Yes | No | No | No | 13 | Yes | 36.6 | 3.9 | 2.8 | 2.7 |
| 115 | 2 | male | 3 | Yes | Yes | No | No | No | No | Yes | 15 | Yes | 3.6 | 4.3 | 4.1 | 4.3 |
| 116 | 3 | male | 2 | Yes | No | Yes | Yes | Yes | Yes | Yes | 40 | No | 1.9 | 4.0 | 2.3 | 2.1 |
| 117 | 1 | male | 4 | Yes | No | Yes | No | No | No | No | 36 | No | 1.6 | 4.5 | 1.7 | 1.8 |
| 118 | 2 | female | 3 | Yes | Yes | Yes | No | No | No | No | 32 | Yes | 1.8 | 3.7 | 2.2 | 2.3 |
| 119 | 3 | female | 2 | Yes | Yes | No | No | No | No | No | 25 | No | 1.8 | 4.1 | 2.3 | 2.4 |
| 120 | 3 | female | 2 | Yes | No | Yes | No | No | No | No | 26 | No | 1.4 | 2.2 | 2.3 | 2.4 |
| 121 | 4 | male | 4 | Yes | Yes | No | Yes | No | No | No | 5 | Yes | 4.3 | 2.5 | 2.5 | 2.2 |
| 122 | 2 | male | 3 | No | No | Yes | No | Yes | No | No | 29 | No | 3.2 | 4.3 | 3.6 | 3.5 |
| 123 | 2 | female | 1 | No | No | No | Yes | No | No | No | 30 | No | 2.9 | 4.2 | 2.8 | 2.9 |
| 124 | 4 | male | 3 | No | Yes | No | No | Yes | No | Yes | 32 | Yes | 1.1 | 3.2 | 1.6 | 1.4 |
| 125 | 4 | male | 2 | No | Yes | Yes | Yes | Yes | No | Yes | 11 | Yes | 2.1 | 3.5 | 2.3 | 2.6 |
| 126 | 3 | female | 2 | Yes | No | No | No | No | No | No | 27 | No | 1.0 | 3.4 | 1.3 | 1.4 |
| 127 | 4 | female | 2 | Yes | Yes | No | No | No | No | Yes | 6 | Yes | 10.2 | 2.7 | 1.0 | 5.4 |

CN: copy number
